# The Eurasian shrew and vole tick *Ixodes trianguliceps*: geographical distribution, climate preference, and pathogens detected

**DOI:** 10.1007/s10493-023-00797-0

**Published:** 2023-05-09

**Authors:** Franz Rubel, Olaf Kahl

**Affiliations:** 1 Unit for Veterinary Public Health and Epidemiology, University of Veterinary Medicine Vienna, Veterinärplatz 1, 1210 Vienna, Austria; 2 tick-radar GmbH, Berlin, Germany

**Keywords:** Distribution maps, Köppen–Geiger climate classification, Small mammals, Rodents

## Abstract

The Eurasian shrew and vole tick *Ixodes trianguliceps* Birula lives in the nests and burrows of its small mammalian hosts and is—along with larvae and nymphs of *Ixodes ricinus* or *Ixodes persulcatus*—one of the most commonly collected tick species from these hosts in its Eurasian range. *Ixodes trianguliceps* is a proven vector of *Babesia microti*. In this study, up-to-date maps depicting the geographical distribution and the climate preference of *I. trianguliceps* are presented. A dataset was compiled, resulting in 1161 georeferenced locations in Eurasia. This data set covers the entire range of *I. trianguliceps* for the first time. The distribution area between 8$^\circ$ W–105$^\circ$ E and 40–69$^\circ$ N extends from Northern Spain to Western Siberia. To investigate the climate adaptation of *I. trianguliceps*, the georeferenced locations were superimposed on a high-resolution map of the Köppen–Geiger climate classification. The Köppen profile for *I. trianguliceps*, i.e., a frequency distribution of the tick occurrence under different climates, shows two peaks related to the following climates: warm temperate with precipitation all year round (Cfb), and boreal with warm or cold summers and precipitation all year round (Dfb, Dfc). Almost 97% of all known *I. trianguliceps* locations are related to these climates. Thus, *I. trianguliceps* prefers climates with warm or cold summers without dry periods. Cold winters do not limit the distribution of this nidicolous tick species, which has been recorded in the European Alps and the Caucasus Mountains up to altitudes of 2400 m. Conversely, *I. trianguliceps* does not occur in the Mediterranean area with its hot and dry summers.

## Introduction

**Fig. 1:**
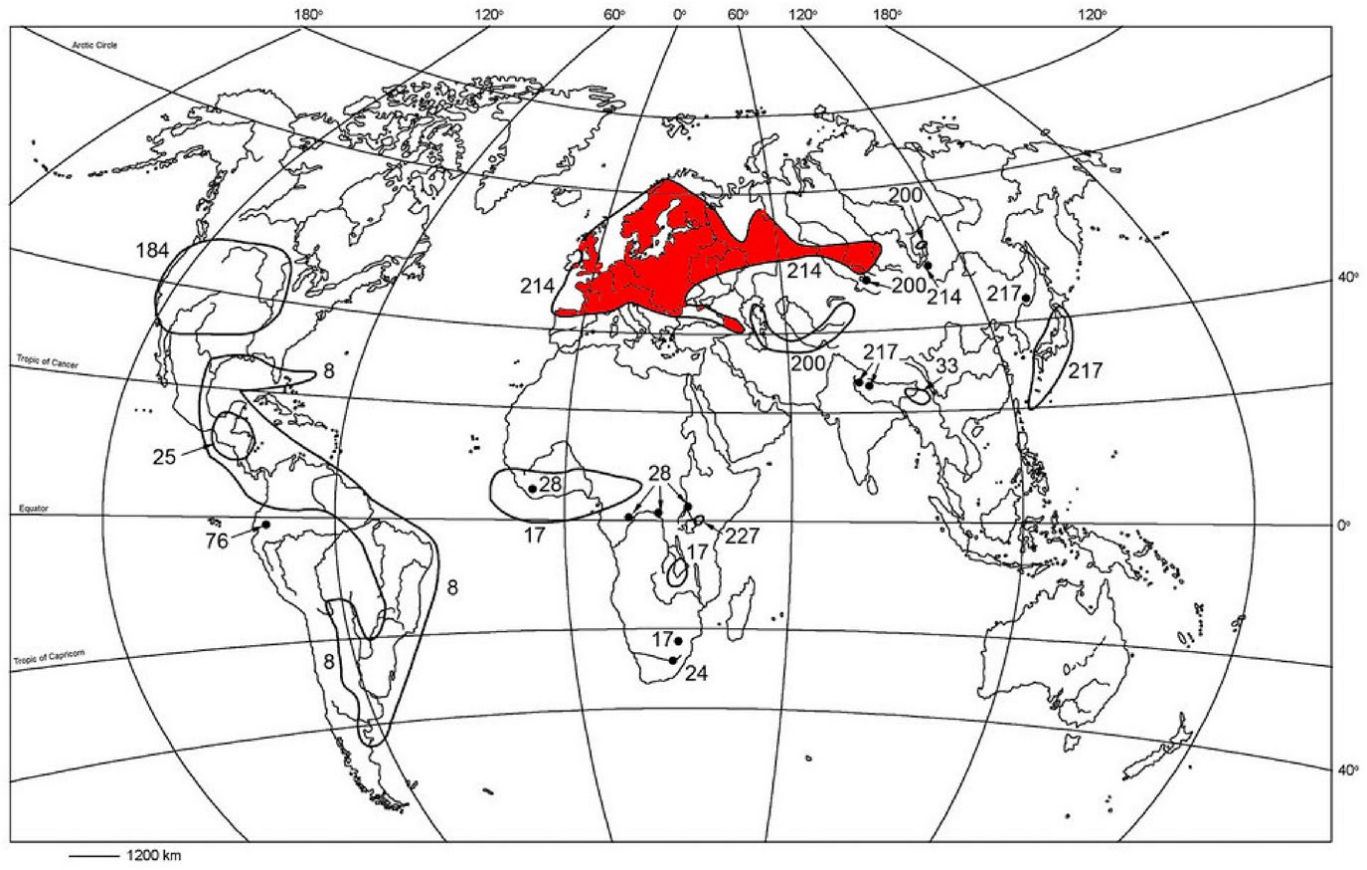
Global geographical distribution of *Ixodes trianguliceps* (red area, 214), adapted and coloured from the no longer available online tick atlas by Kolonin (2009). Contour lines and dots provided with different numbers indicate the distribution of other tick species not used here

*Ixodes trianguliceps* Birula, the Eurasian shrew and vole tick (Acari, Ixodidae), is a proven vector of *Babesia microti* (Young 1970; Hussein 1980; Randolph 1995). It is a three-host tick species of the subgenus *Exopalpiger* Schulze, endemic in wide areas of Europe and Asia (Filippova 2010). In many countries such as the United Kingdom (Cotton and Watts 1967), France (Morel 1965) and Russia (Sapegina 1967), *I. trianguliceps* has been investigated for a long time. An early global distribution map with 45 tick locations was already presented by O’Donnell (1973). Figure 1 depicts the geographical distribution of the tick species as published by Kolonin (2009). It has been the most complete distribution map for *I. trianguliceps* presented so far, showing a main range of 9$^\circ$ W–88$^\circ$ E and an isolated occurrence at 105$^\circ$ E. The often quoted online tick atlas of Kolonin (2009) is unfortunately no longer available, so reference is made here also to the earlier map by Kolonin (1981). Other distribution maps are restricted to national territories such as the *I. trianguliceps* maps of the former Soviet Union (Korenberg and Lebedeva 1969), Switzerland (Graf et al. 1979), former Yugoslavia (Tovornik 1988), as well as Great Britain and Ireland (Martyn 1988). More recent maps, which also take historical findings into account, have been compiled for Germany (Rubel et al. 2021, 2023) and Austria (Rubel and Brugger 2022). A list of all countries with *I. trianguliceps* reports was very recently compiled by Guglielmone et al. (2023).

All postembryonic life stages of *I. trianguliceps* infest mainly burrowing small mammals. It colonizes moist (not wet) habitats in deciduous, mixed and coniferous forests. In Russia, most *I. trianguliceps* findings are located within the dark-coniferous forest of the Central and Southern Taiga. It is less often found in pine, broad-leaved and aspen-birch forests. Occasionally, the tick penetrates into the Northern Taiga and the forest-steppe (Korenberg and Lebedeva 1969). *Ixodes trianguliceps* also occurs at high altitudes above the treeline. In the European Alps (Aeschlimann et al. 1970; Mahnert 1971) and the Caucasus Mountains (Filippova and Stekolnikov 2007) the tick has been found up to an altitude of 2400 m. The tick was also found in the high mountain areas of Sweden and Norway, where the northernmost location is documented north of the Arctic Circle on the Lofoten Islands (Nilsson 1974). These occurrences indicate that *I. trianguliceps* is a rather cold-resistant *Ixodes* species, but it must be emphasized that its typical off-host habitat in the soil seems to be well protected from bad frost. Only two other tick species, namely the seabird tick *Ixodes uriae* (Munoz-Leal and González-Acuna 2015) and the castor bean tick *Ixodes ricinus* have been recorded at these northern latitudes of Scandinavia.

Research into the biology of *I. trianguliceps* began in the 1950s and early 1960s (Vysotskaya 1951; Nikitina 1960; Lachmajer 1962). It is a nidicolous tick, i.e., living in small mammals’ dens and burrows, where it might have rather easy access to its hosts. It has mainly been found on *Apodemus sylvaticus* mice and *Myodes glareolus* voles in the United Kingdom (Bown et al. 2003), on *Sorex araneus*, *Sorex alpinus*, *Sorex minutus* shrews, *Apodemus flavicollis* mice, as well as on *Microtus agrestis* and *Microtus nivalis* voles in Austria (Mahnert 1971). Some other small mammals such as the European dormouse *Glis glis* and the European mole *Talpa europaea* (Tovornik 1988) have also been mentioned as host species. The 53 host species in Russia listed by Korenberg and Lebedeva (1969) aditionally include less common hosts such as the red fox *Vulpes vulpes*, ground-feeding birds such as the mistle thrush *Turdus viscivorus* and reptiles such as the common lizard *Lacerta vivipara*.

It should be noted that the global distribution of *I. trianguliceps* corresponds quite well to that of one of its most important hosts, the bank vole *Myodes glareolus* (formerly *Clethrionomys glareolus*), whose distribution area can be retrieved from the International Union for Conservation of Nature (2022). *Ixodes trianguliceps* is also considered a rare, accidental parasite of humans (Pfäffle et al. 2017). Although all life stages of *I. trianguliceps* appear to be active, i.e., feeding on hosts, throughout the year, there is a distinct annual cycle (Cotton and Watts 1967; Mahnert 1971; Ulmanen 1972) with most adult ticks being found from April to May. The highest activity of the nymphs was mostly observed from June to August. Larvae have a bimodal activity with a first peak in spring and a second peak in autumn. However, the seasonal activity of *I. trianguliceps* is subject to strong variation depending on both the climatic region and the weather of the respective year. For example, the activity peaks of all tick stages in the warm southwest of France (Gilot et al. 1976a) occur much earlier than in the higher altitudes of the European Alps (Mahnert 1971) or the higher latitudes of Scandinavia (Ulmanen 1972).

Digital world maps (Kottek et al. 2006; Rubel and Kottek 2010) and high-resolution maps for the European Alps (Rubel et al. 2017) of the Köppen–Geiger climate classification were used here to investigate the climate adaptation of *I. trianguliceps*. This most widespread climate classification goes back to a cooperation between the German–Russian meteorologist Wladimir Köppen and the German climatologist Rudolf Geiger (Köppen 1936; Geiger 1961). Global maps of the Köppen–Geiger climate classification have been used to characterize the suitable climate for *Ixodes scapularis* (Feria-Arroyo et al. 2014), *Argas miniatus* and *Argas persicus* (Muñoz-Leal et al. 2018), *Haemaphysalis concinna* (Rubel et al. 2018), as well as *Dermacentor reticulatus* and *Dermacentor silvarum* (Rubel et al. 2020).

In this paper new maps depicting the complete geographical distribution of *I. trianguliceps* as it is known to date are presented, to relate georeferenced tick sampling sites to a global climate classification.

## Materials and methods

Since there was no data set on the global distribution of *I. trianguliceps* in Eurasia, a comprehensive literature search was carried out. For this purpose, the authors have used their personal literature collection, which has been built up over decades. It contains historical works going back to 1854, mostly in German, English, French, Italian, Spanish and Russian, and has been regularly updated with new publications via PubMed, Scopus and Google Search. This data set on the distribution of ticks in Europe and the adjacent areas of Asia and Africa was therefore not created through a systematic review specifically for this paper, but in the classic way through many years of expert work. It refers mainly to that kind of literature in which georeferenced findings are documented. For example, geographical coordinates of *I. trianguliceps* locations are already available for Austria (Rubel and Brugger 2022), Belgium (Obsomer et al. 2013) and Germany (Rubel et al. 2014, 2021, 2023). Digital coordinates for Great Britain and Ireland (Martyn 1988) were archived by the National Biodiversity Network (2022), and a collection of Swiss locations (Graf et al. 1979) can be obtained from the Centre Suisse de Cartographie de la Faune (2022). However, the Swiss locations were taken from the original publication. In order to close data gaps, however, tick findings were also digitized if sufficient text information on the locations or printed maps were available. According to Table 1 the following numbers of *I. trianguliceps* locations were incorporated: 3 in Armenia, 12 in Austria, 8 in Belgium, 8 in Bulgaria, 5 in the Czech Republic, 16 in Croatia, 5 in Estonia, 22 in Finland, 94 in France, 46 in Germany, 290 in Great Britain and Ireland, 3 in Hungary, 8 in Italy, 4 in Lithuania, 2 in the Netherlands, 6 in Norway, 15 in Poland, 4 in Romania, 52 in Russia, 73 in the Scandinavian countries, 4 in Serbia, 5 in Slovakia, 261 in the former Soviet Union, 8 in Spain, 5 in Sweden, 73 in Switzerland, 2 in Turkey, 7 in Ukraine, and 116 in former Yugoslavia.

As depicted in Table 1, most references considered describe observations from the period 1960–2000. Although there are also many publications after the year 2000, they only contain a few *I. trianguliceps* findings. Despite this, much effort has been expended to map these recent *I. trianguliceps* findings as they may confirm older occurrences. Importantly, large parts of Eurasia are not adequately covered by available studies. Thus, the handdrawn map by Korenberg and Lebedeva (1969) was digitized, without which a good coverage of the countries of the former Soviet Union would not have been possible. The same applies to the Balkans, for which the tick locations from former Yugoslavia (Tovornik 1988) were digitized. The location on the Crimea was taken from the map by O’Donnell (1973).

Digitized locations, of course, are generally of lower accuracy than locations described by geographical coordinates determined by GPS in the field. To provide evidence of this, accuracy measures were given for all data referenced in Table 1 in accordance with a scheme established in previous studies. It is distinguished between high (h $\approx$ 0.1 km), medium (m $\approx$ 1 km), low (l $\approx$ 10 km) and unspecified (u) accuracies. The latter has been applied here only to the German (Rubel et al. 2014, 2021, 2023) and Austrian (Rubel and Brugger 2022) records that contain tick locations of all accuracy levels.

To visualize the geographical distribution of *I. trianguliceps*, the georeferenced locations were plotted on terrain maps (OpenStreetMap contributors 2017). They show the distribution patterns of the tick determined by continental-scale mountain ranges like the Himalayas and surrounding steppes and deserts. The latter were also depicted in a second type of maps, where the tick locations were plotted on climate maps. Therefore, updated global maps of the Köppen–Geiger climate classification (Rubel and Kottek 2010) were used. Generally, the Köppen–Geiger climate classification is based on 31 climate classes described by a three-letter code. The first letter distinguishes between different types of vegetation of the equatorial zone (A), the arid zone (B), the warm temperate zone (C), the boreal or snow zone (D), and the polar or ice zone (E). The second letter in the classification considers precipitation (e.g., Cf for warm temperate and precipitation all year round) and the third letter considers air temperature (e.g., Cfb warm temperate, precipitation all year round and warm summer).

The climate map (version December 2018) is provided on https://koeppen-geiger.vu-wien.ac.at together with the underlying digital data and an R code (R Development Core Team 2022) for reading and visualization. The gridded climate classification is available with a spatial resolution of 5 arcmin and representative for the 25-year period 1986–2010. It was calculated from downscaled, i.e., disaggregated (Rubel et al. 2017), temperature and precipitation fields as described by Kottek et al. (2006). With this dataset, each tick location can be related to a specific climate class in order to calculate a histogram. Recent applications of this so-called Köppen profile were, for example, presented by Grímsson et al. (2018) and Rubel et al. (2018, 2020).

Finally, the literature search included the occurrence of microorganisms or their DNA/RNA in *I. trianguliceps* ticks and also research on the vector competence of *I. trianguliceps* for any pathogens. Because this tick species is endophilous and it is usually not possible to collect its unfed stages by flagging, infections of *I. trianguliceps* with microorganisms have been detected only in individuals removed from hosts. However, this approach leaves it open whether the found microorganisms were freshly taken by that tick with the current bloodmeal or whether the unfed tick had already carried that infection. As a consequence, even a positive result leaves the critical eco-epidemiological question open whether or not *I. trianguliceps* is a vector of the found microorganism.

## Results and discussion

**Fig. 2:**
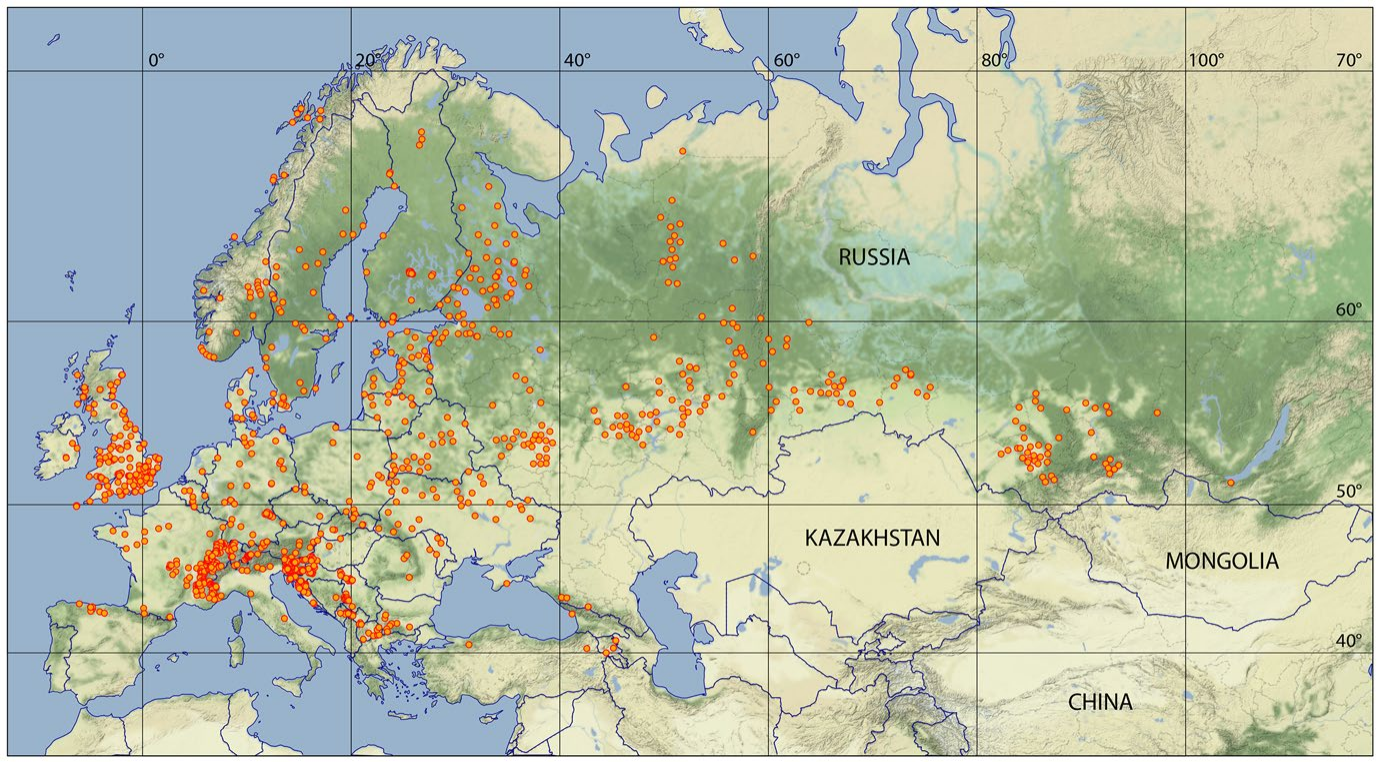
Findings of *Ixodes trianguliceps* (orange dots) ranging between 8$^\circ$ W–105$^\circ$ E and 40–69$^\circ$ N

**Fig. 3:**
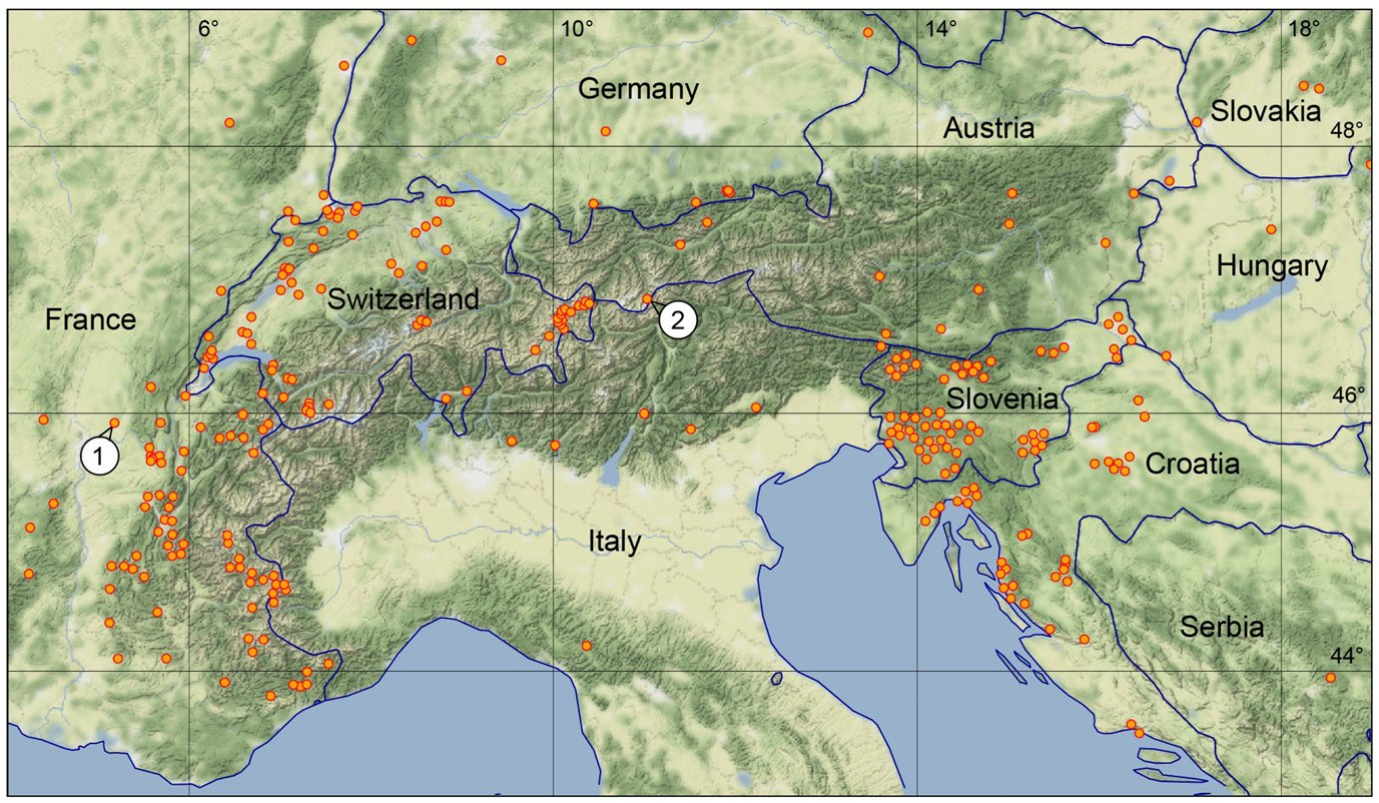
Findings of *Ixodes trianguliceps* in the Greater Alpine Region (GAR), centered at 11.5$^\circ$ E/44.5$^\circ$ N. The marked *I. trianguliceps* locations near Lyon, France (1) and Obergurgl, Austria (2) are discussed in the text

Figure 2 depicts a map of the entire distribution areas of the shrew and vole tick *I. trianguliceps* and a higher resolution section of the Greater Alpine Region (GAR) is shown in Fig. 3. The GAR map was chosen to demonstrate the preferred occurrence of *I. trianguliceps* in cooler climate regions such as the European Alps. There the tick has been found both in Switzerland at Göscheneralp (Aeschlimann et al. 1970) and in Austria at Obergurgl (Mahnert 1971) up to an altitude of 2300 m. In the Czech Republic, *I. trianguliceps* was found in the High Tatras near the Téry cottage at an altitude of 2016 m (Cerny 1959) and in the Russian Caucasus region at Mt. Elbrus up to an altitude of 2400 m (Filippova and Stekolnikov 2007). The tick was also found in the high mountain areas of Sweden and Norway, where the northernmost location is documented on the Lofoten Islands at 68.7$^\circ$ N (Nilsson 1974). The southernmost location was documented in Turkey at 40.3$^\circ$ N (Keskin and Selcuk 2021). In the Balkans, the occurrence of *I. trianguliceps* has also been documented down to southern latitudes of 41$^\circ$ N (Tovornik 1988). The distribution area of *I. trianguliceps* is thus in the latitude belt of 40–69$^\circ$ N. In southern Europe, this is about five degrees of latitude south of the southernmost limit shown in Fig. 1. This map adapted from Kolonin (1981, 2009) dates from before 1980, when the author apparently had no information about the occurrence of *I. trianguliceps* in the Balkans, in the Italian Apennines and on the western Turkish Black Sea coast. East of the Caucasus, however, the southern distribution limit is consistently ten degrees of latitude further north. The southernmost observation of *I. trianguliceps* in its Siberian distribution range is also the easternmost location documented near Lake Baikal at about 105$^\circ$ E/51$^\circ$ N (Vershinina 1988). With the westernmost *I. trianguliceps* findings in Ireland at 7.3$^\circ$ W (Martyn 1988) the global distribution can be estimated. Thus, the documented distribution area extends from Ireland/Northern Spain to Western Siberia between 8$^\circ$ W–105$^\circ$ E and 40–69$^\circ$ N. However, the documented locations of *I. trianguliceps* are unevenly distributed within this area. Clustered tick occurrences or even data gaps are mainly due to the presence or absence of regional field studies and should not be interpreted biologically.

**Fig. 4:**
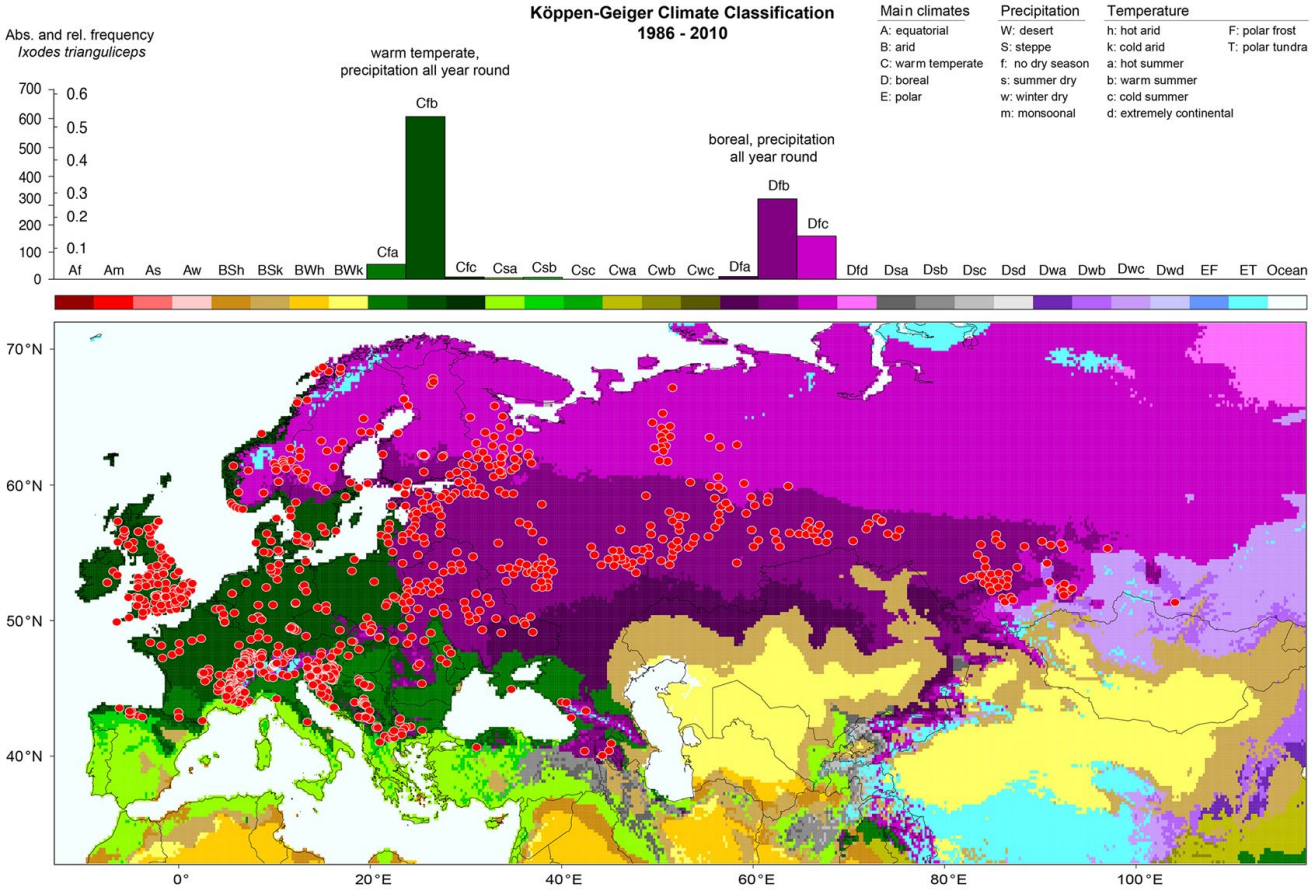
Findings of *Ixodes trianguliceps* superimposed on the map of the Köppen-Geiger climate classification (defined by a three-letter code and representative for the period 1986–2010) and frequency distribution of *I. trianguliceps* occurrence. Absolute frequencies depict the number of tick locations, relative frequencies the fraction of tick locations in each climate class. Highest frequencies of *I. trianguliceps* occurrence were observed in warm temperate climates with precipitation all year round (Cfb) and boreal (continental) climates with precipitation all year round (Dfb, Dfc), both with warm or cold summers

A key result is the determination of the climate preference of *I. trianguliceps* crucial for its global distribution. For this purpose, the tick locations were superimposed on the Köppen–Geiger climate classification map and a frequency distribution of these tick locations in different climate zones was compiled. Figure 4 shows the climate classification map together with the Köppen profile for *I. trianguliceps*. The latter shows a histogram of the frequency of tick findings reported for different climate classes. Two peaks are related to the following climates: warm temperate with precipitation all year round Cf (58%) and boreal with precipitation all year round Df (41%). Thus, a total of 99% of all *I. trianguliceps* locations was reported in these climates, and it is evident that *I. trianguliceps* prefers precipitation all year round. This agrees surprisingly well with the Köppen profile for *Dermacentor reticulatus* (Rubel et al. 2020), a tick species with which *I. trianguliceps* is sympatric in large parts of its range. Due to its nidicolous off-host life, however, *I. trianguliceps* is even better ecologically adapted to cold, which is why it also occurs at higher altitudes and at higher geographical latitudes than *D. reticulatus*. The below-ground microclimate in the host burrows is not identical with that above ground. This is important to bear in mind when talking about climate adaptation in the following. Macroclimatic temperature extremes are attenuated below-ground (and also below snow in the winter).

**Fig. 5:**
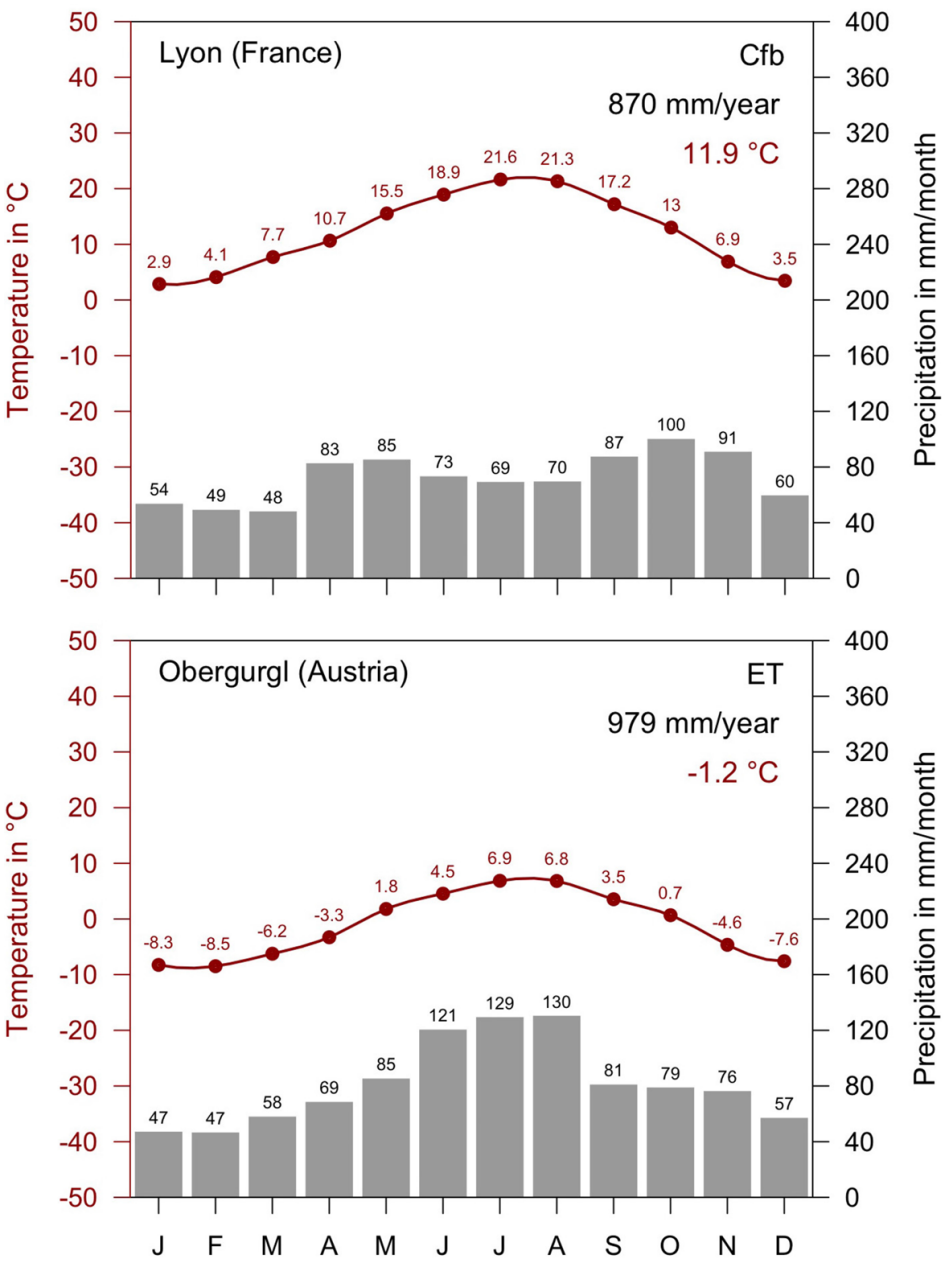
Climate diagrams for the period 1986–2010. Warm temperate climate with year-round precipitation and warm summers (Cfb) in Lyon, France, typical of more than 50% of the recorded *Ixodes trianguliceps* locations, and Alpine tundra climate (ET) in Obergurgl, Austria, reflecting the macroclimatic cold adaptation of *I. trianguliceps* at 1920 m altitude

The two high alpine *I. trianguliceps* findings near Göscheneralp and Obergurgl described above are located in the so-called Alpine belt above the tree and forest line. This altitudinal belt—for details see Rubel et al. (2017)—is characterized by the tundra climate ET, whose lower limit is defined by the 10 $^\circ$C isotherm. This means that the maximum monthly mean temperature is below 10 $^\circ$C, such as in Obergurgl with a July mean temperature of 6.9 $^\circ$C (Fig. 5). As at all other *I. trianguliceps* locations, precipitation falls in Obergurgl all year round with an annual precipitation of 979 mm/year. During the winter months, precipitation falls as snow, resulting in about 130 days of snow cover per year (Koch et al. 2020). The mean annual temperature is negative at −1.2 $^\circ$C. However, it can be assumed that *I. trianguliceps* ticks tolerate even more extreme macroclimatic conditions than those shown in the climate diagram (Fig. 5), since the study area of Mahnert (1971) was 400 m above Obergurgl. There, the snow cover is present for about 150 days a year. A second climate diagram from Lyon, France, shows the significantly warmer Cfb climate, in which 52% of the *I. trianguliceps* findings collected here are located (Fig. 5). In Lyon, a mean annual temperature of 11.9 $^\circ$C and a mean annual precipitation of 870 mm/year were observed in the period 1986–2010. Generally, the warm temperate Cfb climate is defined for a temperature range of $-\,3 \,^{\circ }$C $< T_{min} < + 18^{\circ }$ C, a maximal monthly temperature of $T_ {max} < 22\,^{\circ }$C, and at least four months with $T_{mon} \ge 10\,^{\circ }$C (Kottek et al. 2006).

Less than one percent of the *I. trianguliceps* findings are just outside the climate classes discussed. This can be caused by imprecise georeferenced tick findings, insufficient spatial resolution of the climate data or a temporal discrepancy between the tick findings and the climate data. Thus, the southern distribution in Europe is limited by the Mediterranean climate, characterized by the summer-dry climates Csa and Csb. *Ixodes trianguliceps* definitely does not occur in the Mediterranean region (light green in Fig. 4). In southern Siberia, the hot summers of the Dfa climate, which is replaced further south by the steppe climate BSk, apparently limit the spread of *I. trianguliceps*. In the east, the distribution of *I. trianguliceps* is limited by the winter-dry climates Dwb and Dwc, where the cold can enter the soil much more easily without a buffering snow cover. If one considers the shift in climate zones observed since 1900 and projected up to the year 2100 (Rubel and Kottek 2010), the distribution area of *I. trianguliceps* might have changed only insignificantly in the past and only small changes are to be expected for the future. There could potentially be population declines in northern France and the Balkans if those regions do indeed get warmer and drier summers. However, it must be considered that although current climate models can predict the temperature well, changes in the precipitation regime are subject to great uncertainty.

At this point it should be noted that all *I. trianguliceps* findings described in the literature were subjected to a plausibility check in the present study. As a result, a total of four locations in Iran (Hamidi and Bueno-Mar 2021) has been excluded from the data set, as also practiced by Guglielmone et al. (2023). These findings, with mean coordinate of 59$^\circ$ E/36$^\circ$ N, are far south of the known distribution area described above. In addition, Hamidi and Bueno-Mar (2021) state that *I. trianguliceps* was collected only from the Persian jird *Meriones persicus*, which inhabits dry, rocky slopes with sparse vegetation and steppe. In fact, two of the locations are in the Mediterranean climate Csa and two in the steppe climate BSk. It can thus be assumed that the *I. trianguliceps* ticks reported from Iran were misidentifications. In contrast, ticks collected from migratory birds in eastern Poland were included, since they occur in the natural range of *I. trianguliceps*. At the Kaliszany Ornithological Station, *I. trianguliceps* were collected from blackcaps *Sylvia atricapilla* and song thrushes *Turdus philomelos* (Zajac et al. 2022).

Table 2 provides a summary of the pathogens found in *I. trianguliceps* removed from hosts. A possible role of *I. trianguliceps* in natural foci of tick-borne encephalitis (TBE) and hemorrhagic fever renal syndrome (HFRS), which motivated the early work of Korenberg and Lebedeva (1969), was not confirmed. There is currently no study that found TBE virus in *I. trianguliceps*, and it is now known that HFRS is caused by hanta viruses, which are transmitted through aerosolized excrement of rodents. However, numerous pathogenic bacteria and protozoa have been found in *I. trianguliceps*. It must be pointed out that the finding of any tick-borne pathogens in ticks removed from hosts is no proof of vector competence. Without proven capability of transmission the vector function of a given tick species for a given pathogen is not substantiated (Kahl et al. 2002). Transmission studies with *I. trianguliceps* are only available for *Babesia microti*, which were carried out for the first time by Young (1970) in the United Kingdom. Based on this, Randolph (1995) quantified the parameters of the natural transmission cycle of *B. microti* between the tick vector *I. trianguliceps* and the host *M. glareolus*. In experimental infection studies, transovarial transmission of *B. microti* could be ruled out and the natural transmission cycle explained by host-to-vector, vector-to-vector, and vector-to-host transmission. Without experimental transmission carried out, but indicated by extensive field studies, *I. trianguliceps* is a putative vector for *Anaplasma phagocytophilum* (Bown et al. 2003, 2008). The same applies to the repeated finding of *Borrelia burgdorferi* s.l. spirochaetes in *I. trianguliceps* removed from hosts. They would also justify experimental transmission studies, but for the time being this tick species can only be called a possible vector of *B. burgdorferi* s.l. (Eisen 2020).

## Conclusions and outlook

To summarize the current knowledge of the distribution of *I. trianguliceps*, a dataset of 1161 locations was collated to compile a geographical map covering its whole distribution range from Ireland and the Spanish Atlantic coast in the west to Lake Baikal in the east. Although there are numerous recent studies on *I. trianguliceps* (Keskin and Selcuk 2021; Mysterud et al. 2015; Obert et al. 2015; Tretyakov 2017), field studies are totally lacking in some regions or were carried out many decades ago, so that there is also potential for improving the map presented here. As already demonstrated for other tick species (Rubel et al. 2018, 2020), all known locations of *I. trianguliceps* were assigned climate classes using digital data from the global Köppen–Geiger climate classification (Kottek et al. 2006). The result is a very clear climate profile for *I. trianguliceps*, according to which the tick species occurs primarily in the warm temperate and boreal climate zones with precipitation in all seasons. However, *I. trianguliceps* has also occasionally been found in Alpine tundra climates. Looking at the list of pathogens found in feeding *I. trianguliceps* ticks (Table 2) and the only experimental transmission studies concerning *B. microti*, it becomes clear that there is a great need for having more such studies to prove or disprove its vector competence for some tick-borne pathogens (Bonnet and Nadal 2021) in order to support public health authorities.

The here presented data might be a suitable basis for compiling maps based on species distribution models as already applied for *Dermacentor reticulatus* (Brugger and Rubel 2023). The latter are part of a set of digital tick maps projected onto the virtual globe Google Earth. These Google Earth maps are currently being developed in order to offer scientists from various disciplines, but also the interested public, simple and modern access to tick distribution maps. The first maps of the distribution of *I. trianguliceps* have already been compiled and can be downloaded as a preview from the following link https://epidemic-modeling.vetmeduni.ac.at/.

**Table 1:** Number, accuracy (low, medium, high and unspecified), and country of georeferenced *Ixodes trianguliceps* sampling sites compiled in this study

No.	Acc.	Country	References
3	l	Armenia	Dilbaryan and Hovhannisyan (2016)
12	u	Austria	Rubel and Brugger (2022)
8	h	Belgium	Obsomer et al. (2013)
8	l	Bulgaria	Pfäffle et al. (2017)
3	l	Caucasian Countries	Filippova and Stekolnikov (2007)
4	l	Czech Republik	Cerny (1959)
1	l	Czech Republik	Hubálek et al. (1990)
16	l	Croatia	Krčmar (2012)
5	h	Estonia	Vikentjeva et al. (2021)
6	l	Finland	Ulmanen (1972)
16	h	Finland	Cayol et al. (2018)
11	l	France	Rageau (1972)
67	l	France	Gilot et al. (1976a, 1976b, 1979)
4	l	France	Morel (1965)
4	l	France	Doby et al. (1990)
2	l	France	L’Hostis et al. (1996)
4	l	France	Boyard et al. (2008)
1	h	France	Pisanu et al. (2010)
1	l	France	Perez et al. (2017)
46	u	Germany	Rubel et al. (2014, 2021, 2022, 2023)
290	l	Great Britain & Ireland	Martyn (1988)
3	l	Hungary	Janisch (1959)
4	l	Italy	Manilla (1990)
1	l	Italy	Flavioni (2016)
1	h	Italy	Morini et al. (2018)
1	l	Italy	Baráková et al. (2018)
1	l	Italy	Martello et al. (2019)
4	l	Lithuania	Paulauskas et al. (2010)
2	h	Netherlands	van Duijvendijk et al. (2022)
1	h	Norway	Mysterud et al. (2015)
3	h	Norway	Hvidsten et al. (2020)
2	h	Norway	De Pelsmaeker et al. (2021)
1	m	Poland	Siuda et al. (2009)
5	l	Poland	Haitlinger (2010)
3	l	Poland	Izdebska and Kadulski (2011)
1	l	Poland	Solarz et al. (2010)
4	l	Poland	Nowak-Chmura (2013)
1	l	Poland	Zajac et al. (2022)
1	l	Romania	Coipan et al. (2011)
3	l	Romania	Mihalca et al. (2012)
20	l	Russia	Lutta (1968)
17	l	Russia	Zolotov et al. (1974)
2	l	Russia	Vershinina (1988)
1	l	Russia	Balashov et al. (2003)
1	l	Russia	Kerbabaev and Tsushba (2011)
1	h	Russia	Kolchanova and Bragina (2011)
5	l	Russia	Obert et al. (2015)
1	m	Russia	Kormilitsyna et al. (2016)
3	l	Russia	Rar et al. (2016)
1	l	Russia	Starikov et al. (2017)
1	h	Russia	Tretyakov (2017)
1	l	Russia	Bespyatova et al. (2019)
2	l	Russia	Sabitova et al. (2023)
73	l	Scandinavian Countries	Nilsson (1974)
4	l	Serbia	Petrović et al. (2016)
2	l	Slovakia	Nosek et al. (1967)
2	h	Slovakia	Blanarová et al. (2014)
1	l	Slovakia	Svitálková et al. (2015)
257	l	Soviet Union	Korenberg and Lebedeva (1969)
1	l	Soviet Union	O’Donnell (1973)
7	l	Spain	Gilot et al. (1976c)
1	l	Spain	Dominguez (2014)
5	l	Sweden	Grandi et al. (2023)
72	l	Switzerland	Graf et al. (1979)
1	l	Switzerland	Eichenberger et al. (2015)
2	l	Turkey	Keskin and Selcuk (2021)
6	l	Ukraine	Naglova and Naglov (1983)
1	l	Ukraine	Nebogatkin (2015)
116	l	Yugoslavia	Tovornik (1988)
**1161**	–	**Total**

**Table 2:** Detection of tick-borne pathogens or their DNA/RNA in *Ixodes trianguliceps* removed from host

Pathogen bacteria	Disease	Country	References
*Anaplasma phagocytophilum*	Granulocytic anaplasmosis	England	Bown et al. (2003, 2006)
	in humans and animals	Russia	Kolchanova and Bragina (2011)
		Netherlands	Jahfari et al. (2014)
		Slovakia	Blanarová et al. (2014)
*Borrelia burgdorferi* s.l.	Lyme borreliosis	France	Doby et al. (1990)
		Russia	Grigoryeva and Tretyakov (1998)
*B. afzelii*		Russia	Korenberg et al. (2015)
*B. garinii*		Russia	Korenberg et al. (2015)
*B. bavariensis*		Russia	Sabitova et al. (2023)
*Candidatus* B. sibirica		Russia	Sabitova et al. (2023)
*Ehrlichia chaffeensis*	Monocytotropic ehrlichiosis	Russia	Kolchanova and Bragina (2011)
*E. muris*	Monocytotropic ehrlichiosis	Russia	Kolchanova and Bragina (2011)
*Francisella tularensis*	Tularaemia	Slovakia	Guryčová (1998)
		Russia	Kormilitsyna et al. (2016)
*Candidatus* Neoehrlichia mikurensis	Neoehrlichiosis	Slovakia	Blanarová et al. (2016)
*Rickettsia helvetica*	Rickettsiosis	Russia	Igolkina et al. (2015)
*Candidatus* R. tarasevichiae		Russia	Igolkina et al. (2015)
*Candidatus* R. uralica		Russia	Igolkina et al. (2015)
		Estonia	Vikentjeva et al. (2021)
Piroplasmorida (Protozoa)			
*Babesia microti*	Babesiosis	England	Hussein (1980)
		Germany	Obiegala et al. (2015)
		Russia	Rar et al. (2016)
